# FECH Expression Correlates with the Prognosis and Tumor Immune Microenvironment in Clear Cell Renal Cell Carcinoma

**DOI:** 10.1155/2022/8943643

**Published:** 2022-08-25

**Authors:** Guanghui Zhong, Qing Li, Yang Luo, Yufeng Liu, Dawei Liu, Bin li, Tao Wang

**Affiliations:** Department of Urology, The Fifth Affiliated Hospital, Southern Medical University, Guangzhou, Guangdong 510900, China

## Abstract

**Background:**

Clear cell renal cell carcinoma (ccRCC) is, by far, the most prevalent and fatal kind of kidney cancer. Ferrochelatase (FECH) is an enzyme that performs a significant function in the onset and progression of many distinct kinds of malignant tumors. Nevertheless, its predictive usefulness in renal clear cell carcinoma (RCC) has not yet been fully investigated.

**Methods:**

FECH expression in ccRCC and healthy adjoining tissues was primarily screened utilizing data sourced from The Cancer Genome Atlas (TCGA) and subsequently validated using data from an independent cohort derived from the Gene Expression Omnibus (GEO) and the Human Protein Atlas HPA databases. The relationship among FECH expression, clinicopathological parameters, and overall survival (OS) was assessed utilizing multivariate analysis and Kaplan–Meier survival curves. Additionally, the protein networks with FECH interaction were constructed with the aid of the online Search Tool for the Retrieval of Interacting Genes/Proteins (STRING). Gene ontology (GO) analysis, and gene set enrichment analysis (GSEA) were conducted based on TCGA data, and a single-sample GSEA was utilized to explore the link between FECH expression and the infiltration status of immune cells in the tumor. The Gene Expression Profiling Interactive Analysis (GEPIA) and TIMER databases were utilized to investigate the relationships of FECH expression with the infiltrating immune cells and the matching gene marker sets.

**Results:**

FECH expression was shown to be substantially lowered in ccRCC tumors as opposed to that observed in normal tissues (*p* < 0.05). Lower levels of FECH expression were shown to have a strong association with higher grades of cancer and more advanced TNM stages. The findings of multivariate and univariate analyses illustrated that the OS in patients with ccRCC with low FECH expression is shorter in contrast with that in the high FECH expression group (*p* < 0.05). It was discovered that CPOX and frataxin are key proteins that interact with FECH. ccRCC with FECH deficiency was linked to the lack of infiltrating immune cells and their respective marker sets, which included CD4+ T cells.

**Conclusion:**

In ccRCC, decreased FECH expression was linked to disease progression, unfavorable prognosis, and impaired immune cell infiltration.

## 1. Introduction

The number of people diagnosed with renal cell carcinoma (RCC) has been growing steadily over the last several decades all over the globe. In particular, RCC is ranked first among urological tumors with respect to the annual mortality rate [1]. RCC is a heterogeneous tumor, with clear cell renal cell carcinoma (ccRCC) constituting roughly 75%–80% of RCCs [2]. ccRCC is distinguished from other cancers by the early-stage disappearance of the von Hippel–Lindau tumor-suppressor gene expression in the majority of tumors [3, 4]. Presently, the standard therapy used to treat ccRCC is targeted therapy; nonetheless, nearly all patients eventually deteriorate as ccRCC cells escape drug-induced apoptosis or autophagy [5]. Ferroptosis is a unique kind of cell death, and its induction is gaining popularity as a potentially viable therapeutic option for ccRCC [6–9]. However, current therapies only work in a subset of patients, and it is imperative to identify more effective therapeutic targets for ccRCC. Furthermore, the discovery of additional biological markers that might aid in early diagnosis and lead to an improvement in prognosis is a very necessary endeavor.

Ferrochelatase, also known as FECH, is an enzyme that performs a critical function in catalyzing the process of transforming protoporphyrin IX (PpIX) to heme. The heme biosynthesis pathway, which is present in all the cells, is responsible for many crucial aspects of cell metabolism, which include oxygen transport, the modulation of cellular oxidation, and the metabolism of drugs [10]. In the process that leads to the formation of heme, the enzyme, aminolevulinic acid (ALA) synthase, first acts as a rate-limiting enzyme. It is responsible for the synthesis of 5-aminolevulinic acid (5-ALA), which is subsequently fixed via the heme biosynthesis pathway, ultimately resulting in the formation of PpIX. PpIX is a fluorescence-emitting molecule that serves as the direct precursor of heme. PpIX fluorescence may be utilized for the photodynamic detection of malignancies owing to the mechanism that cancer cells produce large amounts of PpIX in response to treatment with exogenous 5-ALA [11–13]. Moreover, irradiating PpIX at certain wavelengths causes the release of reactive oxygen species (ROS), which ultimately results in the death of cancer cells. The term for this kind of treatment is photodynamic therapy (5-ALA-PDT) [14]. Oncogenic transformation is generally believed to promote the accumulation of 5-ALA-elicited PpIX in cancer cells. Additionally, oncogenic transformation upregulates certain enzymes in the heme biosynthesis pathway, such as porphobilinogen synthase, coproporphyrinogen-III oxidase (CPOX), and porphobilinogen deaminase, all of which speed up the production of PpIX [15–17]. Thus, reduced FECH expression can lead to PpIX accumulation in tumor cells under treatment with exogenous 5-ALA. Kemmner et al. reported the significant downregulation of FECH mRNA expression in rectal, colon, and gastric cancers. Furthermore, the knockdown of FECH expression with small interfering RNA (siRNA) technology resulted in a maximum increase of 50-fold in PpIX buildup, which might be feasible using specially manufactured equipment for two-photon microscopy [18].

In this study, we investigated the relationship among FECH expression, clinical information, and OS of ccRCC patients by analyzing data retrieved from various databases, namely, the GEO, TCGA, and HPA. Following this, we collected data from the TIMER and GEPIA databases to examine the link between FECH expression and infiltration of immune cells and the associated gene marker sets. Additionally, the FECH-interacting protein network was evaluated with the help of the STRING website. A low FECH level was connected with reduced infiltrating immune cells in ccRCC tissues, which served as an indication of a dismal prognosis. Hence, a defect in FECH expression may increase PpIX accumulation and possibly attenuate the antitumor immune impacts in ccRCC. FECH-related targeting may be a viable treatment approach in ccRCC along with/in combination with immunotherapy.

## 2. Materials and Methods

### 2.1. Data Source

TCGA (https://portal.gdc.cancer.gov), a publicly available data platform for a large-scale cancer genome project, offers clinicopathological data on 33 distinct kinds of cancer and is easily accessible to researchers and academics. The TCGA database was searched for clinical data related to patients diagnosed with ccRCC as well as high-throughput RNA sequencing (RNA-seq) information. The fragments per kilobase per million fragments mapped (FPKM) approach that is included in HTSeq was used to determine the levels of transcript expression. In addition, for subsequent investigation, the RNA-seq gene expression level 3 HTSeq-FPKM information of 539 patients suffering from ccRCC and the accompanying clinical data were transformed into the format of transcripts per million (TPM) reads.

The GEO database, which encompasses one of the world's biggest compilations of gene chips, is a complete and comprehensive gene expression resource at the National Center for Biotechnology Information (https://www.ncbi.nlm.nih.gov/geo/). The gene expression profile data were downloaded from two GEO datasets (GSE66271 and GSE53757) by GEOquery package, and then, the differences between the two groups were analyzed by limma package. Because the database is public, no permission from the local ethics committee was necessary.

### 2.2. The HPA Databases

The HPA offers substantial data regarding the transcriptome and proteome of various human specimens, encompassing tissue, cell, and pathology Atlas. Currently, this Web-based database encompasses data on the cell-specific positions for 44 normal tissues as well as twenty of the most frequently diagnosed cancers. Moreover, the database also provides data on protein immunohistochemical in tumors and normal human tissues.

### 2.3. Clinical Statistical Examination of Prognosis, Model Development, and Assessment

Analyses of prognostic parameters, such as OS, disease-specific survival (DSS), and progression-free interval, were performed in the clinical meaning module of the Xiantao platform (https://www.xiantao.love/) premised on the patient data derived from the TCGA. These analyses were executed utilizing the Cox regression and Kaplan–Meier techniques. The median value was employed to determine the cutoff value of low- and high-FECH expression groups. To ascertain the connection between clinicopathological characteristics and FECH expression, we utilized the Wilcoxon signed-rank sum test in conjunction with logistic regression. The influence of FECH expression on the chance of survival and other clinical variables was investigated with the use of a multivariate Cox regression model. The threshold for significance was established at a *P*-value less than 0.05. The findings from the Cox regression model were utilized in conjunction with the independent prognostic variables acquired from the multivariate analysis, and survival rates over 1, 3, and 5 years were anticipated using these data. Through calibration curves, the anticipated rates were compared with the actual occurrences that took place. The 45-degree line represented the extreme accuracy of the predicted value.

### 2.4. Protein-Protein Interaction (PPI) Comprehensive Analysis

The STRING web platform (https://string-db.org/) was also adapted for data analysis. The website provides extensively integrated and consolidated PPI data. Following the importation of the FECH expression data into the STRING platform, we retrieved the information on the PPI network. The significance threshold was set at a confidence score greater than 0.7.

### 2.5. Enrichment Analysis

The gene ontology (GO) enrichment analysis of FECH expression was executed with the help of R's clusterProfiler program (version 3.6.3) and included the analyses of molecules with differential expression, namely, those under cellular components (CC), molecular functions (MF), and biological processes (BP). The settings were adjusted as follows: enrichment factor >1.5, minimum count >3, and *P* < 0.01. The GSEA [19] approach was utilized to rank the genome a thousand times for each study and enrich pathways associated with FECH expression. In the GSEA, the cutoff value for statistically meaningful findings was determined to be an adjusted *P* < 0.05 and a false discovery rate (FDR) of <0.25. The outcomes of the enrichment analysis were defined by utilizing the normalized enrichment scores (NESs) and adjusted *P*-values. The GSEA and visualization were both performed with the help of the Cluster Profiler tool [20].

### 2.6. Analysis of the Infiltration of Immune Cells

A research report that was published by Bindea et al. [21] was consulted to acquire the marker genes for each of the 24 distinct types of immune cells. The ssGSEA approach was utilized to investigate the infiltration of the tumor with twenty-four different kinds of immune cells. The Spearman correlation algorithm was utilized not only for the assessment of infiltration levels of immune cells between high- and low-FECH expression groups but also for the assessment of the strength of association between FECH expression and the infiltration levels of the 24 distinct kinds of immune cells. The link between FECH expression and immune infiltration as well as the association between infiltration levels of immune cells and the values obtained in various FECH expression groups were analyzed in the module of the “Xiantao tool” based on the findings of immune infiltration, Xiantao tool Spearman correlation, and Wilcoxon signed-rank sum.

### 2.7. Gene Correlation Analysis

GEPIA (http://gepia.cancer-pku.cn/index.html) is a web platform that offers information on 9736 different types of cancers as well as 8587 normal specimens derived from TCGA and GTEx. It focuses on the analysis of the findings of the RNA-seq. The Gene Classes and the Isoform Classes each specify the kinds of the corresponding number of types of genes and isoforms, which come to a total of 60,498 and 198,619, correspondingly. In the GEPIA database, an investigation was conducted to determine the nature of the connection that exists between the expression of FECH and a variety of immune cell markers. The level of expression of the FECH is shown along the *x*-axis, whereas the expression of other tested genes is displayed along the *y*-axis. Additionally, we verified the expression of genes that exhibited a strong link to FECH expression in GEPIA premised on data from TIMER (http://cistrome.org/TIMER/).

## 3. Results

### 3.1. FECH Expression Was Decreased in Tumors as Opposed to Normal Samples

**Figure 1:**
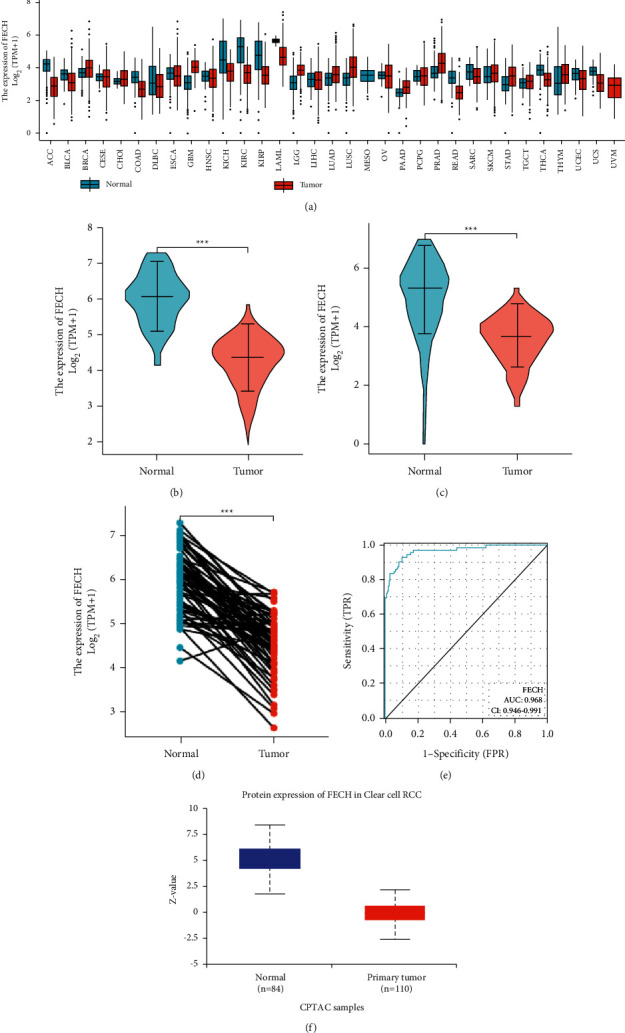
Status of FECH expression in malignancies. (a) Profile of FECH expression in distinct human tumors and homologous healthy tissues. (b) Differences between FECH expression in KIRC tissues and adjacent healthy tissues. (c) Variations between FECH expression in normal samples (obtained using GTEx data) and adjoining ccRCC tissues and ccRCC samples. (d) Variations between FECH expression in ccRCC samples and corresponding adjoining samples. (e) ROC curve for FECH expression in normal samples (obtained using GTEx data) and adjoining ccRCC tissues and ccRCC samples. (f) FECH protein expression was considerably downregulated in tumor tissues in contrast with that in nonpaired normal tissues (^*∗*^*P* < 0.05, ^*∗∗*^*P* < 0.01, and ^*∗∗∗*^*P* < 0.001).

**Figure 2:**
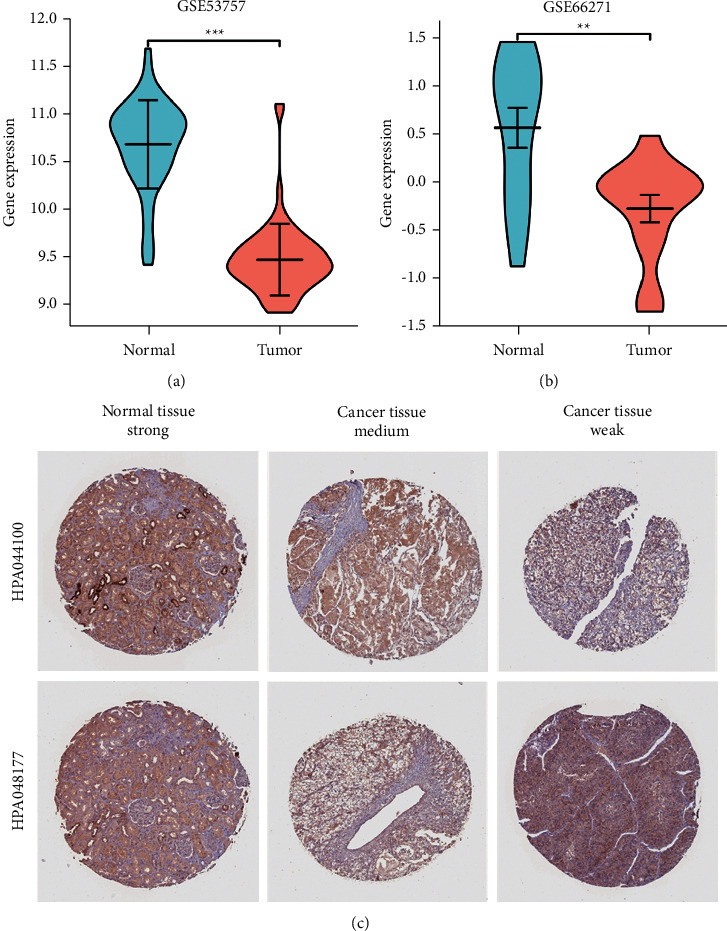
Assessment of FECH gene expression in GEO datasets and the HPA data. (a) Verification of the decreased expression of FECH mRNA in ccRCC relative to normal tissues in the GSE53757 dataset. (b) Verification of the decreased expression of FECH mRNA in ccRCC relative to normal samples in the GSE66271 dataset. (c) FECH protein expression in renal cell carcinoma tissue was attenuated in contrast with that in normal tissue in the HPA data (antibodies HPA044100 and HPA048177,10X) (^*∗*^*P* < 0.05, ^*∗∗*^*P* < 0.01, and ^*∗∗∗*^*P* < 0.001).

To determine whether low FECH expression in cancer is a generalized phenomenon, we began by analyzing the FECH expression pan-cancer and compared it with that in the corresponding adjacent healthy tissues in the TCGA dataset (Figure 1(a)). The information included in the TCGA database was utilized to make predictions about the profiles of FECH mRNA expression in 539 ccRCC and 72 normal samples (Figure 1(b)). The FECH mRNA expression in ccRCC primary tumor specimens was remarkably attenuated in contrast with those in normal tissues (*P* < 0.001). Additionally, we examined FECH expression in normal specimens (data obtained from GTEx) in comparison with adjoining ccRCC tissues and that of ccRCC samples and discovered that FECH expression was downmodulated in ccRCC tissues (*P* < 0.001) (Figure 1(c)). Moreover, FECH expression was substantially downmodulated in 72 ccRCC samples in contrast with corresponding adjoining samples (*P* < 0.001) (Fig.  1(d)). Subsequently, a receiver operating characteristic (ROC) curve was charted to examine the diagnostic significance of FECH expression by performing a comparison between FECH expression in normal specimens (data obtained from GTEx) and adjoining ccRCC tissues with that of ccRCC samples. The findings illustrated that the area under the curve (AUC) value for FECH levels was 0.968 (CI = 0.946–0.991), indicative of a strong potential for diagnostic application (Figure 1(e)). The degree of FECH protein expression was likewise downmodulated in ccRCC tissues contrasted with that in normal specimens (Figure 1(f)). This indicates that the protein and mRNA expression patterns of FECH were comparable across various databases. In addition, the level of FECH gene expression was checked for accuracy in the GEO datasets (GSE66271 and GSE53757) (Figure 2(a) and 2(b)). Correspondingly, using the data derived from the HPA, the expression of FECH protein was shown to be downmodulated in ccRCC tissue as opposed to that in normal tissue (Figure 2(c)).

### 3.2. Association of FECH Expression with Clinical Parameters

**Figure 3:**
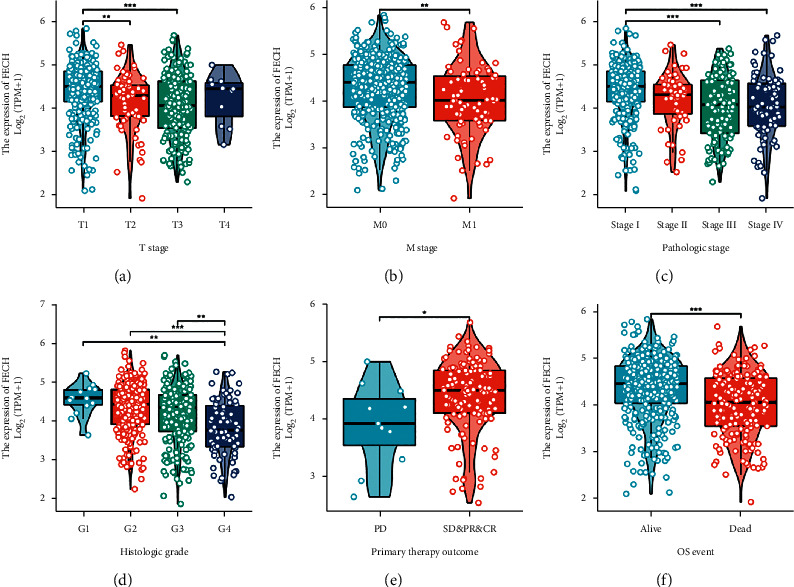
Association between FECH expression and clinicopathological parameters of ccRCC. Association between FECH expression and T stage (a), M stage (b), pathological stage (c), histological stage (d), primary therapeutic outcome (e), and overall survival event (f). (^*∗*^*P* < 0.05, ^*∗∗*^*P* < 0.01, ^*∗∗∗*^*P* < 0.001).

The proportion of FECH expression in tumor specimens was measured with the aid of the Z-score criterion, and the ccRCC cohort was then classified into low- and high-expression groups premised on the levels of FECH expression. To ascertain the connection between FECH expression and clinical parameters, we utilized the Kruskal–Wallis test as well as the Wilcoxon signed-rank test. A lower level of FECH expression was reported in cases of higher *M* stage, *T* stage, and pathological stages and also in cases of higher histological grade and OS events (*P* < 0.05, Figure 3(a)–3(f)). Concurrently, similar findings were obtained after conducting the Fisher's exact test or the chi-squared test (Table 1). Besides, a strong association between FECH expression and clinical parameters, particularly pathological grade, was also shown by the findings of the univariate analysis of FECH expression [odds ratio (OR) = 0.407 (0.284–0.582), *P* < 0.001], histological grade [odds ratio (OR) = 0.600 (0.425–0.845), *P*=0.004], *T* stage [OR = 0.435 (0.301–0.623), *P* < 0.001], and *M* stage [OR = 0.501 (0.300–0.820), *P*=0.007] (Table 2). However, we did identify any statistically meaningful difference in the association with the N stage [OR = 1.078 (0.385–3.019), *P*=0.885], age [OR = 0.856 (0.610–1.199), *P*=0.366], and gender [OR = 0.882 (0.618–1.258), *P*=0.488] (Table 2). Based on these findings, the expression of FECH was connected to the clinical features in ccRCC.

### 3.3. Prognostic Relevance of FECH Expression in ccRCC

**Figure 4:**
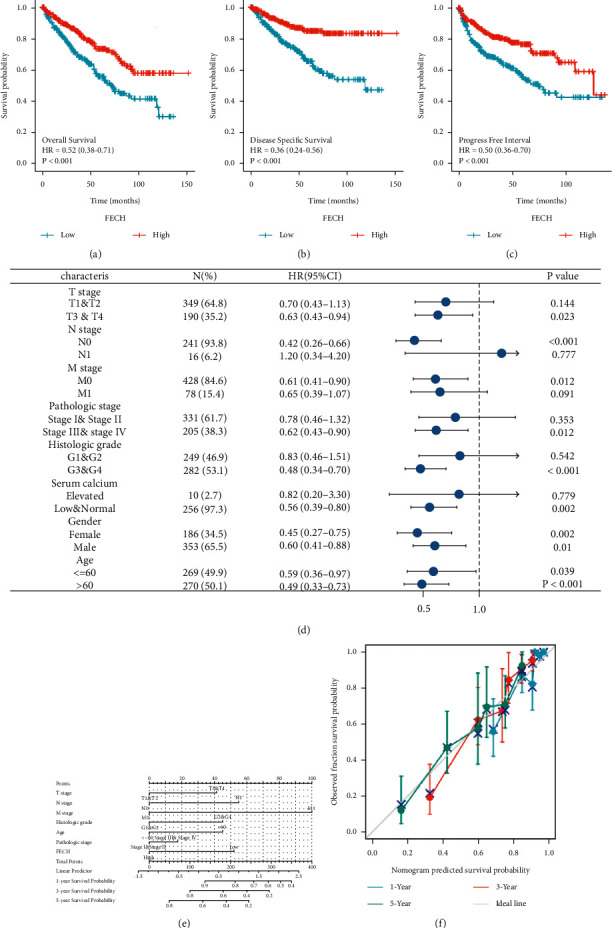
FECH expression prognostic analysis. Patients who had low FECH expression had unfavorable prognosis indicators as opposed to patients whose FECH expression was high, including shorter overall survival (OS) (a), progression-free interval (PFS) (b), and disease-specific survival (DSS) (c) (both log-rank *P* < 0.001). (d) Prognosis based on FECH expression in distinct kinds of clinical features (OS). (e) Nomogram for multivariate analysis premised on clinical features linked to FECH expression. (f) The prediction accuracy of the model that was determined via the use of multifactor Cox regression analysis is displayed in the calibration chart.

Figures 4(a)–4(c) show that premised on the information sourced from the TCGA database, the links between FECH expression and prognosis indicators (OS, DSS, and PFS). There was a correlation between low FECH expression and unfavorable OS [hazards ratio (HR) = 0.52 (0.38–0.71), *P* < 0.001, Figure 4(a)], unfavorable DSS [HR = 0.36 (0.24–0.56), *P* < 0.001, Figure 4(b)], and unfavorable PFS [HR = 0.50 (0.36–0.70), *P* < 0.001, Figure 4(c)]. According to the findings, individuals with ccRCC exhibited elevated risk scores and low levels of FECH expression, whereas those with low-risk scores exhibited significant levels of FECH expression. Furthermore, the association between FECH expression and the various groups was investigated in this research. FECH expression was found to be low in the T3-T4 stage [HR = 0.63 (0.43–0.94), *P*=0.023], pathological grade III-IV [HR = 0.62 (0.43–0.90), *P*=0.012], and histological grade G3-G4 [HR = 0.48 (0.34–0.70), *P* < 0.001] (Figure 4(d)). *M* stage, pathological grade, N stage, histological grade, age, *T* stage, and FECH expression were utilized to generate a clinical prognostic risk score for ccRCC (Figure 4(e). Concurrently, with the use of a calibration chart, we evaluated how accurate the model's predictions were (Figure 4(f)). The FECH expression might provide a more accurate prediction of patients' survival chances over 3 and 5 years. Overall, the FECH expression was shown to correlate with the prognosis of individuals diagnosed with ccRCC.

### 3.4. Constructing PPI Networks

**Figure 5:**
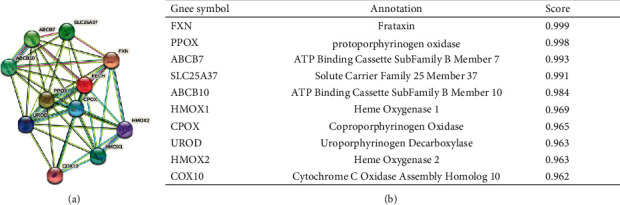
Proteins interacting with FECH in ccRCC tissue. Annotation of proteins that interact with FECH (a), along with their respective co-expression scores (b).

To clarify the molecular basis and metabolic processes involved in malignancy, it is vital to have a good grasp of the functional interaction that takes place between proteins. In order to establish the protein interactions involved in the advancement of ccRCC, an analysis of the PPI network of FECH was performed utilizing the STRING program. In Figure 5, the topmost ten proteins are presented, together with the related gene names, scores, and annotations, including FXN, PPOX, ABCB7, SLC25A37, ABCB10, HMOX1, CPOX, UROD, HMOX2, and COX10.

### 3.5. Expression of FECH in Relation to the Expression Pattern of Whole Genes

**Figure 6:**
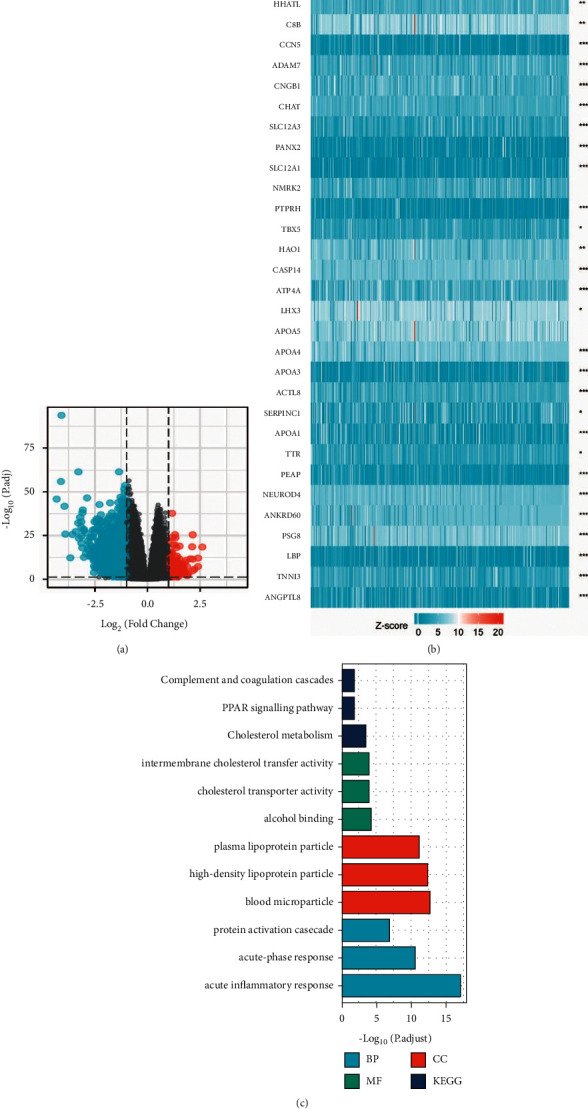
FECH gene expression differential expression and GO enrichment analysis. (a) A volcano map illustrating the differentially expressed genes (DEGs) premised on the FECH expression patterns. (b) The expression level of the FECH gene was used to generate a heat map that displays 30 genes that were either upmodulated or downmodulated. (c) The GO enrichment findings of DEGs that were filtered depending on FECH expression were analyzed via the usage of the Metascape database.

To get a deeper comprehension of the biological role played by FECH in ccRCC, an assessment of the FECH gene expression profile was performed. It was shown that the expression of 3805 genes that were in a downmodulated state and 171 genes that were in an upmodulated state were substantially linked to FECH expression (logFC >1 and Padj <0.05) (Figure 6(a)). In addition, the top 30 genes with aberrant expression levels (abslogFC >2 and Padj <0.01) were displayed on the heat map of the gene expression (Figure 6(b)). Moreover, GO enrichment analysis was carried out premised on the results of the FECH expression. The BP primarily associated with the FECH gene were acute inflammatory responses, acute-phase responses, and regulation of protein activation cascade, among others (Table 3, Figure 6(c)).

### 3.6. GSEA Analysis of FECH Expression

**Figure 7:**
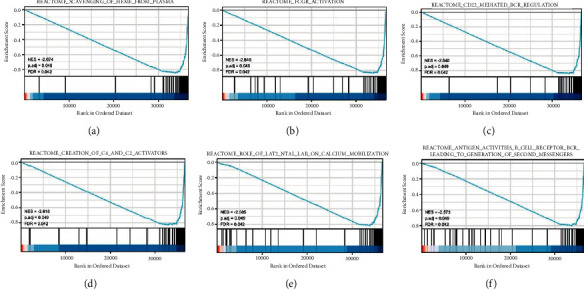
The findings of the GSEA enrichment analysis.

Gene expression data derived from TCGA were subjected to GSEA to determine biological and functional pathways between high- and low-FECH expression groups. The enrichment signaling pathway that was determined to be the most relevant with regard to FECH gene expression was chosen depending on the NESs (Figure 7). The findings of the GSEA analysis illustrated that the low FECH expression phenotype was predominantly concentrated in REACTOME_SCAVENGING_OF_HEME_FROM_PLASMA (A), REACTOME_FCGR_ACTIVATION (B), REACTOME_CD22_MEDIATED_BCR_REGULATION (C), REACTOME_CREATION_OF_C4_AND_C2_ACTIVATORS (D), REACTOME_ROLE_OF_LAT2_NTAL_LAB_ON_CALCIUM_MOBILIZATION (E), and REACTOME_ANTIGEN_ACTIVATES_B_CELL_RECEPTOR_BCR_LEADING_TO_GENERATION_OF_SECOND_MESSENGERS (F).

GSEA results showed that REACTOME_SCAVENGING_OF_HEME_FROM_PLASMA (A), REACTOME_FCGR_ACTIVATION (B), REACTOME_CD22_MEDIATED_BCR_REGULATION (C), REACTOME_CREATION_OF_C4_AND_C2_ACTIVATORS (D), REACTOME_ROLE_OF_LAT2_NTAL_LAB_ON_CALCIUM_MOBILIZATION (E), and REACTOME_ANTIGEN_ACTIVATES_B_CELL_RECEPTOR_BCR_LEADING_TO_GENERATION_OF_SECOND_MESSENGERS (F) were enriched primarily in FECH-associated ccRCC. ES, enrichment score; NES, normalized ES; FDR, false discovery rate.

### 3.7. FECH Expression in Relation to Immune Cell Infiltration

**Figure 8:**
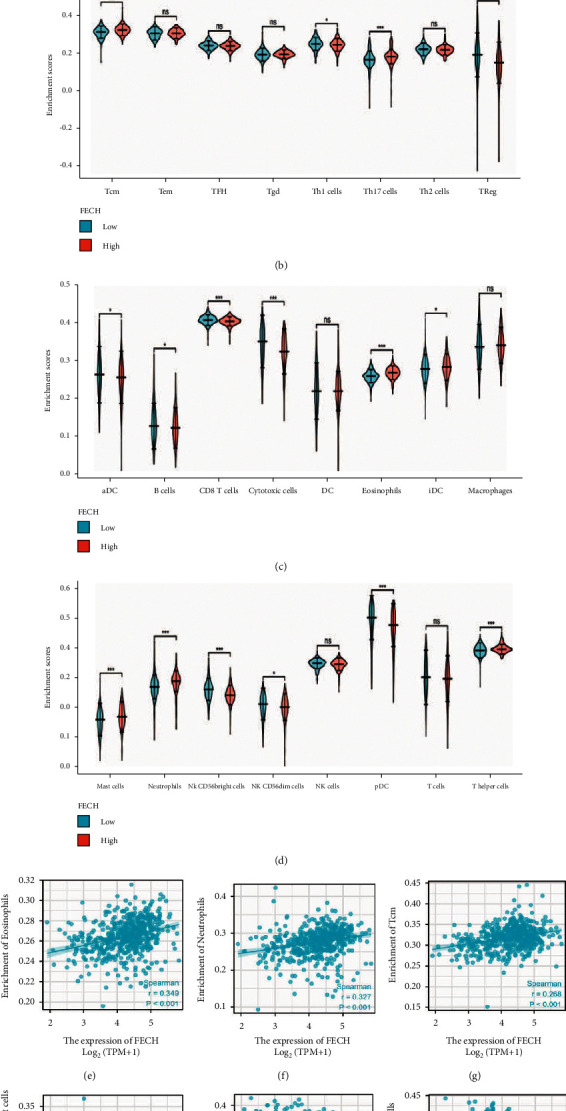
Association of FECH expression with infiltrating immune cells. (a) The connection between the expression of FECH and the infiltration status of immune cells. (b–d) Variations in the degrees to which certain immune cell subsets were enriched in the high- and low-expression FECH groups. (e–j) Associations between FECH expression and tumor microenvironment characteristics. Nonsignificant (ns) denotes *P* > 0.05; ^*∗*^denotes *P* < 0.05; ^*∗∗*^denotes *P* < 0.01; ^*∗∗∗*^denotes *P* < 0.001; ^*∗∗∗∗*^denotes *P* < 0.0001.

Subsequently, the association of FECH expression with 24 distinct immune cell subtypes in ccRCC was investigated and analyzed. FECH expression showed a strong positive link to the infiltration of eosinophils, central memory T cells (T_CM_), neutrophils, and *T* helper cells, and a strong inverse link to the infiltration of NK CD56^bright^ cells, regulatory T cells (Tregs), pDCs, and cytotoxic cells, among others (Figures 8(a), 8(e)–8(j)). Further investigation illustrated substantial differences in the FECH expression level in different infiltrating immune cells such as, aDCs, B cells, iDCs, mast cells, Tregs, TH1 cells, *T* helper cells, and NK CD56^bright^ cells, among others (Figures 8(b)–8(d)). To effectively examine the possible function of FECH in influencing the infiltration status of distinct immune cells in ccRCC, we employed data from the TIMER and GEPIA databases to establish the connection between FECH and different immune marker sets, which are generally acknowledged as being indicators of various immunocytes, comprising DCs, NK cells, M1/M2 macrophages, neutrophils, tumor-associated macrophages (TAMs), B cells, monocytes, T cells (general), and CD8+ T cells, in ccRCC (Table S1). In addition, our research evaluated a range of distinct subtypes of functional T cells, such as Tregs, exhausted T cells, Th1, Th2, Th9, Th17, Th22, and Tfh. According to the findings, the expression of the majority of immune set markers for various types of DCs, M1/M2 macrophages, TAMs, and T cells was shown to be linked to the expression level of FECH in ccRCC.

## 4. Discussion

5-ALA, which is the metabolism precursor of heme in the heme biosynthesis pathway, is not a fluorescence molecule but is instead converted into the endogenously fluorescent compound PpIX [22]. The biosynthesis of heme requires a number of stages to be catalyzed by enzymes, with the final step involving the transformation of PpIX into heme by FECH, which is located within the inner membrane of the mitochondria [18]. Excitation of PpIX takes place when it is subjected to a suitable source of light with a certain wavelength. This enables PpIX to become detectable as a result of the generation of red fluorescence with a bimodal functionality, which serves as both a fluorescent marker and 5-ALA-PDT. PDT is comprised of a chain of photobiological and photochemical processes that pose irreparable damage to cancer cells. When compared to normal brain tissues, glioma tissues underwent a considerable reduction in FECH mRNA expression. This finding leads to the hypothesis that FECH is attributable to the buildup of PpIX in glioma cells. The ablation of FECH in glioma cells by the use of the siRNA approach resulted in increased fluorescence of PpIX, which occurred concomitantly with an increase in the amount of PpIX that accumulated within the cells in response to 5-ALA. In glioma cells that had been treated with PDT, suppressing FECH expression resulted in a remarkable attenuation in growth and an enhancement in the process of apoptosis [23]. FECH is the last enzyme in heme biosynthesis pathway. Inhibited FECH caused iron overload in cancer cells and triggered iron concentration, thereby inhibiting cancer cell growth [24]. In addition, the RAS/MEK pathway increased PpIX accumulation in cancer cells by regulating the FECH activity mechanism, which would facilitate precise recognition of tumor boundaries and small satellite tumors [25]. Relevant studies have shown that changes in FECH expression were detected in human colon cancers and that loss of FECH has a tumor-suppressive effect on colon carcinogenesis in vitro [26], which was a potential tumor-suppressor gene for colon cancer [27]. The FECH gene may be a switch gene involved in the invasiveness of pituitary nonfunctioning adenomas [28].

Researchers have found a correlation between the presence of attenuated FECH expression or molecular defects in FECH expression in cancerous tumors, which include urothelial and colon cancers, and the buildup of PpIX inside of the cells [29,30]. We observed the considerable downmodulation of FECH mRNA expression in ccRCC in contrast with normal tissues based on data sourced from distinct databases, including GEO, TCGA, and the HPA. Compared with ccRCC cases in which FECH expression was higher, those in which FECH expression was lower showed inferior prognosis. Correspondingly, based on their functionally distinct compositions, CPOX, HMOX1, and HMOX2 were identified as proteins that interacted with FECH in ccRCC, as corroborated by the results of STING analysis. Additionally, ROC analysis demonstrated an AUC of 0.968 in the ccRCC diagnosis, which indicates that FECH may be useful as a possible diagnostic biological marker. In addition, an attenuated expression of FECH was shown to have a favorable correlation with progressive clinicopathological features as well as a dismal prognosis. Moreover, according to the findings of the GO enrichment study, FECH is strongly linked to biological processes including acute-phase response, protein activation cascade, acute inflammatory response, and modulation of protein activation cascade. Univariate and multivariate Cox survival analyses by Zijian Tian et al. showed that expression of the FECH gene was independently associated with overall survival in patients with ccRCC [31], laterally validating the credibility of our findings. No previous studies have demonstrated the association of FECH genes with immune cells, and our study innovatively investigated and analyzed the association of FECH expression in ccRCC with 24 different immune cell subtypes. The results suggested that there were significant differences in the expression levels of FECH in different infiltrating immune cells, and FECH expression showed a strong positive link to the infiltration of eosinophils, central memory T cells (T_CM_), neutrophils, and *T* helper cells, and a strong inverse link to the infiltration of NK CD56^bright^ cells, regulatory T cells (Tregs), pDCs, and cytotoxic cells, among others. This has a certain reference function for the follow-up study of other people.

To this day, the prognosis of patients diagnosed with ccRCC has been determined primarily by clinical and histopathologic parameters, for instance, the status of lymph nodes, the pathological stage of the disease, and the histological grade. Different prognostic markers, gene signatures, and prediction algorithms for DSS and OS have been described by several researchers [32–34]. Our research showed that the FECH expression level has a substantial and consistent link to the infiltration levels of *T* helper cells, T_CM_, neutrophils, and eosinophils in ccRCC. Subsequent analysis of infiltrating lymphocyte markers illustrated that the expression of M1 macrophage markers, in particular, IRF5 and NOS2, showed a weak correlation with the expression of FECH, whereas the expression of M2 macrophage markers, which include MRC1, CD16, and MS4A4A, showed modest correlation with FECH expression, which illustrates the potential regulatory function of FECH expression in the polarization of TAMs. We also discovered that the expression of the markers of CD4+ T cells, including CD4, exhibits a positive relationship with FECH expression. CD4+ T cells are highly versatile with multiple functions that perform a fundamental function in the development and maintenance of efficient antitumor immunity and protumor functions [35]. In the tumor microenvironment, CD4+ T cells have been shown to have a role in tumor invasion and advancement [36].

However, this study has some limitations. First, the current study was based on data retrieved from the online database, which could have led to selection bias due to the limited number of samples, so these results need to be further confirmed in multicenter clinical trials and larger prospective studies. Next, we mainly focused on bioinformatics analysis of FECH expression data. We expect that further experimental studies on FECH expression in vitro and in vivo, biological effects, and potential mechanisms on ccRCC cells will be performed, which contribute to the prospective evaluation of bioinformatics analysis results, and explain the possible function of FECH in ccRCC.

## 5. Conclusions

In conclusion, decreased FECH expression is associated with disease progression, poor prognosis, and impaired immune cell infiltration in ccRCC. This study provides a new possible molecular target for ccRCC, which may ultimately lead to personalized therapy targeting the ccRCC patient population and provides new insights into predicting the efficacy of immunotherapy.

## Figures and Tables

**Table 1:** Relationship between FECH expression and clinicopathological characteristics in patients with ccRCC.

Characteristic	Low expression of FECH	High expression of FECH	*P*
n	269	270	

T stage, *n* (%)			<0.001
T1	107 (19.9%)	171 (31.7%)	
T2	42 (7.8%)	29 (5.4%)	
T3	116 (21.5%)	63 (11.7%)	
T4	4 (0.7%)	7 (1.3%)	

N stage, *n* (%)			1.000
N0	125 (48.6%)	116 (45.1%)	
N1	8 (3.1%)	8 (3.1%)	

M stage, *n* (%)			0.009
M0	202 (39.9%)	226 (44.7%)	
M1	50 (9.9%)	28 (5.5%)	

Pathologic stage, *n* (%)			<0.001
Stage I	105 (19.6%)	167 (31.2%)	
Stage II	32 (6%)	27 (5%)	
Stage III	79 (14.7%)	44 (8.2%)	
Stage IV	51 (9.5%)	31 (5.8%)	

Primary therapy outcome, *n* (%)			0.177
PD	8 (5.4%)	3 (2%)	
SD	2 (1.4%)	4 (2.7%)	
PR	1 (0.7%)	1 (0.7%)	
CR	52 (35.4%)	76 (51.7%)	

Gender, *n* (%)			0.547
Female	89 (16.5%)	97 (18%)	
Male	180 (33.4%)	173 (32.1%)	
Race, *n* (%)			0.533
Asian	3 (0.6%)	5 (0.9%)	

Black or African American	32 (6%)	25 (4.7%)	
White	232 (43.6%)	235 (44.2%)	
Age, *n* (%)			0.413
≤60	129 (23.9%)	140 (26%)	
>60	140 (26%)	130 (24.1%)	

Histologic grade, *n* (%)			<0.001
G1	3 (0.6%)	11 (2.1%)	
G2	104 (19.6%)	131 (24.7%)	
G3	103 (19.4%)	104 (19.6%)	
G4	54 (10.2%)	21 (4%)	

Serum calcium, *n* (%)			0.219
Elevated	8 (2.2%)	2 (0.5%)	
Low	106 (29%)	97 (26.5%)	
Normal	85 (23.2%)	68 (18.6%)	

Hemoglobin, *n* (%)			0.038
Elevated	5 (1.1%)	0 (0%)	
Low	147 (32%)	116 (25.3%)	
Normal	94 (20.5%)	97 (21.1%)	

OS event, *n* (%)			<0.001
Alive	158 (29.3%)	208 (38.6%)	
Dead	111 (20.6%)	62 (11.5%)	

Laterality, *n* (%)			0.734
Left	128 (23.8%)	124 (23%)	
Right	140 (26%)	146 (27.1%)	

Age, median (IQR)	61 (54, 70)	60 (50, 69)	0.144

**Table 2:** Logistic regression analysis of FECH expression.

Characteristics	Total (N)	Odds ratio (OR)	*P*-Value
T stage (T3 and T4 vs. T1 and T2)	539	0.435 (0.301–0.623)	<0.001
N stage (N1 vs. N0)	257	1.078 (0.385–3.019)	0.885
M stage (M1 vs. M0)	506	0.501 (0.300–0.820)	0.007
Pathologic stage (Stage III and Stage IV vs. Stage I and Stage II)	536	0.407 (0.284–0.582)	<0.001
Primary therapy outcome (SD and PR and CR vs. PD)	147	3.927 (1.083–18.531)	0.050
Histologic grade (G3 and G4 vs. G1 and G2)	531	0.600 (0.425–0.845)	0.004
Age (>60 vs. ≤60)	539	0.856 (0.610–1.199)	0.366
Gender (male vs. female)	539	0.882 (0.618–1.258)	0.488
Laterality (right vs. left)	538	1.076 (0.767–1.511)	0.670

**Table 3:** Results of gene ontology enrichment analysis.

Ontology	ID	Description	Gene Ratio	Bg Ratio	P value	P. adjust	Q value
BP	GO:0002526	Acute inflammatory response	21/96	220/18670	4.30e−21	6.40e−18	5.33e−18
BP	GO:0006953	Acute-phase response	10/96	47/18670	3.53e−14	2.63e−11	2.19e−11
BP	GO:0072376	Protein activation cascade	12/96	198/18670	4.16e−10	1.55e−07	1.29e−07
BP	GO:2000257	Regulation of protein activation cascade	10/96	116/18670	4.17e−10	1.55e−07	1.29e−07
BP	GO:0006956	Complement activation	11/96	175/18670	1.58e−09	4.71e−07	3.93e−07
CC	GO:0072562	Blood microparticle	15/101	147/19717	1.02e−15	1.56e−13	1.31e−13
CC	GO:0034364	High-density lipoprotein particle	9/101	26/19717	4.90e−15	3.78e−13	3.17e−13
CC	GO:0034358	Plasma lipoprotein particle	9/101	37/19717	1.86e−13	7.18e−12	6.03e−12
CC	GO:1990777	Lipoprotein particle	9/101	37/19717	1.86e−13	7.18e−12	6.03e−12
CC	GO:0032994	Protein-lipid complex	9/101	39/19717	3.15e−13	9.70e−12	8.15e−12
MF	GO:0043178	Alcohol binding	7/94	85/17697	3.35e−07	7.77e−05	6.03e−05
MF	GO:0017127	Cholesterol transporter activity	4/94	18/17697	2.16e−06	1.44e−04	1.12e−04
MF	GO:0120020	Intermembrane cholesterol transfer activity	4/94	18/17697	2.16e−06	1.44e−04	1.12e−04
MF	GO:0120015	Intermembrane sterol transfer activity	4/94	19/17697	2.72e−06	1.44e−04	1.12e−04
MF	GO:0034987	Immunoglobulin receptor binding	6/94	76/17697	3.10e−06	1.44e−04	1.12e−04
KEGG	hsa04979	Cholesterol metabolism	5/38	50/8076	3.19e−06	2.78e−04	2.52e−04
KEGG	hsa03320	PPAR signaling pathway	4/38	78/8076	4.63e−04	0.019	0.017
KEGG	hsa04610	Complement and coagulation cascades	4/38	85/8076	6.42e−04	0.019	0.017
KEGG	hsa04975	Fat digestion and absorption	3/38	43/8076	0.001	0.023	0.021
KEGG	hsa04977	Vitamin digestion and absorption	2/38	24/8076	0.006	0.097	0.088

## Data Availability

The data used to support the findings of this study are included within the article.

## Supplementary Materials

Supplementary Materials Table 1. Correlation analysis between FECH expression and gene markers of immune cells via “correlation” module of GEPIA and TIMER.

## References

[1] Capitanio U., Bensalah K., Bex A. (2019). Epidemiology of renal cell carcinoma. *European Urology*.

[2] Nabi S., Kessler E. R., Bernard B., Flaig T. W., Lam E. T. (2018). Renal cell carcinoma: a review of biologyand pathophysiology. *F1000Res*.

[3] Frew I. J., Moch H. (2015). A Clearer View of the Molecular Complexity of Clear Cell RenalCell carcinoma. *Annual Review of Pathology: Mechanisms of Disease*.

[4] Ricketts C. J., Linehan W. M. (2018). Multi-regional sequencing elucidates the evolution of clear cell Renal cell carcinoma. *Cell*.

[5] Sanchez-Gastaldo A., Kempf E., Gonzalez Del Alba A., Duran I. (2017). Systemic treatment of renal cell cancer: a comprehensive review. *Cancer Treatment Reviews*.

[6] Atkins M. B., Tannir N. M. (2018). Current and emerging therapies for first-line treatment of metastatic clear cell renal cell carcinoma. *Cancer Treatment Reviews*.

[7] Wettersten H. I., Aboud O. A., Lara P. N., Weiss R. H. (2017). Metabolic reprogramming in clearcell renal cell carcinoma. *Nature Reviews Nephrology*.

[8] Miess H., Dankworth B., Gouw A. M. (2018). The glutathione redox system is essential to prevent ferroptosis caused by impaired lipid metabolism in clear cell renal cell carcinoma. *Oncogene*.

[9] Zou Y., Palte M. J., Deik A. A. (2019). A GPX4-dependent cancer cell state underlies the clear-cell morphology and confers sensitivity to ferroptosis. *Nature Communications*.

[10] Heinemann I. U., Jahn M., Jahn D. (2008). The biochemistry of heme biosynthesis. *Archives of Biochemistry and Biophysics*.

[11] Steinbach P., Weingandt H., Baumgartner R., Kriegmair M., Hofstadter F., Knuchel R. (1995). Cellular fluorescence of the endogenous photosensitizer protoporphyrin IX following exposure to 5-aminolevulinic acid. *Photochemistry and Photobiology*.

[12] Filonenko E. V., Kaprin A., Alekseev B. (2016). 5-Aminolevulinic acid in intraoperative photodynamic therapy of bladder cancer (results of multicenter trial). *Photodiagnosis and Photodynamic Therapy*.

[13] Fiorito V., Chiabrando D., Petrillo S., Bertino F., Tolosano E. (2019). The multifaceted role of heme in cancer. *Frontiers in Oncology*.

[14] Cengel K. A., Simone C. B., Glatstein E. P. D. T. (2016). PDT: what’s past is prologue. *Cancer Research*.

[15] Krieg R. C., Fickweiler S., Wolfbeis O. S., Knuechel R. (2000). Cell-type specific protoporphyrin IX metabolism in human bladder cancer in vitro. *Photochemistry and Photobiology*.

[16] Yang X., Palasuberniam P., Myers K. A., Wang C., Chen B. (2016). Her2 oncogene transformation enhances 5-aminolevulinic acidmediated protoporphyrin IX production and photodynamic therapy response. *Oncotarget*.

[17] Krieg R. C., Messmann H., Rauch J., Seeger S., Knuechel R. (2002). Metabolic characterization of tumor cell–specific protoporphyrin IX accumulation after exposure to 5-aminolevulinic acid in human colonic cells. *Photochemistry and Photobiology*.

[18] Kemmner W., Wan K., Rüttinger S. (2008). Silencing of human ferrochelatase causes abundant protoporphyrin-IX accumulation in colon cancer. *The FASEB Journal*.

[19] Subramanian A., Tamayo P., Mootha V. K. (2005). Gene set enrichment analysis: a knowledge-based approach for interpreting genome-wide expression profiles. *Proceedings of the National Academy of Sciences of the U S A*.

[20] Yu G., Wang L. G., Han Y., He Q. Y. (2012). clusterProfiler: an R package for comparing biological themes among gene clusters. *OMICS: A Journal of Integrative Biology*.

[21] Bindea G., Mlecnik B., Tosolini M. (2013). Spatiotemporal dynamics of intratumoral immune cells reveal the immune landscape in human cancer. *Immunity*.

[22] Inoue H., Kajimoto Y., Shibata M. A. (2007). Massive apoptotic cell death of human glioma cells via a mitochondrial pathway following 5-aminolevulinic acid-mediated photodynamic therapy. *Journal of Neuro-Oncology*.

[23] Teng L., Nakada M., Zhao S. G. (2011). Silencing of ferrochelatase enhances 5-aminolevulinic acid-based fluorescence and photodynamic therapy efficacy. *British Journal of Cancer*.

[24] Yang C., Wang T., Zhao Y. (2022). Flavonoid 4, 4’-dimethoxychalcone induced ferroptosis in cancer cells by synergistically activating Keap1/Nrf2/HMOX1 pathway and inhibiting FECH. *Free Radical Biology and Medicine*.

[25] Yoshioka E., Chelakkot V. S., Licursi M. (2018). Enhancement of cancer-specific protoporphyrin IX fluorescence by targeting oncogenic ras/MEK pathway. *Theranostics*.

[26] Safi R., Mohsen-Kanson T., Nemer G. (2019). Loss of ferrochelatase is protective against colon cancer cells: ferrochelatase a possible regulator of the long noncoding RNA H19. *Journal of Gastrointestinal Oncology*.

[27] Kadara H., Nemer G., Safi R. (2017). Erythropoietic protoporphyria a clinical and molecular study from Lebanon: ferrochelatase a potential tumor suppressor gene in colon cancer. *Clinical Genetics*.

[28] Khayer N., Jalessi M., Jahanbakhshi A., Tabib khooei A., Mirzaie M. (2021). Nkx3-1 and Fech genes might be switch genes involved in pituitary non-functioning adenoma invasiveness. *Scientific Reports*.

[29] Peng Q., Warloe T., Berg K. (1997). 5-Aminolevulinic acid-based photodynamic therapy. Clinical research and future challenges. *Cancer*.

[30] Miyake M., Ishii M., Kawashima K. (2009). siRNA-mediated knockdown of the heme synthesis and degradation pathways: modulation of treatment effect of 5-aminolevulinic acid-based photodynamic therapy in urothelial cancer cell lines. *Photochemistry and Photobiology*.

[31] Tian Z., Meng L., Wang X. (2022). CGN correlates with the prognosis and tumor immune microenvironment in clear cell renal cell carcinoma. *Frontiers in Molecular Biosciences*.

[32] Volpe A., Patard J. J. (2010). Prognostic factors in renal cell carcinoma. *World Journal of Urology*.

[33] Meskawi M., Sun M., Trinh Q. D. (2012). A review of integrated staging systems for renal cell carcinoma. *European Urology*.

[34] Patard J. J., Kim H. L., Lam J. S. (2004). Use of the University of California Los Angeles integrated staging system to predict survival in renal cell carcinoma: an international multicenter study. *Journal of Clinical Oncology*.

[35] Fridman W. H., Pages F., Sautes-Fridman C., Galon J. (2012). The immune contexture in human tumours: impact on clinical outcome. *Nature Reviews Cancer*.

[36] Xiang P., Jin S., Yang Y. (2019). Infiltrating CD4+ T cells attenuate chemotherapy sensitivity in prostate cancer via CCL5 signaling. *The Prostate*.

