# Supplementary material for: FECH Expression Correlates with the Prognosis and Tumor Immune Microenvironment in Clear Cell Renal Cell Carcinoma
Source: J Oncol. 2022 Aug 25;2022:8943643. doi: 10.1155/2022/8943643 (PMC9436586; doi:10.1155/2022/8943643)
Supplement: Supplementary Materials — Table 1. Correlation analysis between FECH expression and gene markers of immune cells via “correlation” module of GEPIA and TIMER. [file 8943643.f1.pdf]

TableS1. Correlation analysis between FECH expression and gene markers of Immune cells via "correlation" module of GEPIA and TIMER.

Description	markers	Tumor		Normal		None		Purity	
		R	P value	R	P value	R	P value		
CD8+ T cell	CD8B	_0.0055	0.9	_0.4	0.0005	-0.084	0.053	-0.07	0.134
	CD8A	0.024	0.58	_0.49	1.1e_05	-0.0357	0.411	-0.0197	0.673
B cell	CD19	_0.022	0.61	_0.39	0.00079	-0.184	1.84029E-05	-0.16	0.000560036
	KRT20	0.028	0.53	_0.17	0.14	-0.109	0.012	-0.099	0.034
	CD38	0.053	0.23	_0.22	0.061	0.093	0.032	0.108	0.021
Th1	TBX21	0.078	0.074	_0.36	0.0021	0.015	0.737	0.033	0.474
	IFNG	0.014	0.74	_0.29	0.013	-0.084700382	0.051	-0.068050148	0.144613716
	STAT1	0.32	1.6e_13	_0.028	0.82	0.30281437	1.18E-12	0.320863146	1.69E-12
	IL12RB2	0.15	0.00066	_0.078	0.51	0.101510617	0.019072527	0.102917192	0.02713315
	IL27RA	_0.0051	0.91	_0.19	0.11	-0.066178157	0.126999755	-0.029391816	0.529027149
	STAT4	0.0026	0.95	_0.44	0.00013	-0.03999123	0.356804944	-0.004202113	0.928304194
	TNF-α	0.16	0.00026	_0.18	0.12	0.075122049	0.083148227	0.090577881	0.051955014
Th2	GATA3	_0.024	0.59	0.44	0.00013	-0.090444216	0.03687734	-0.052133954	0.263958286
	STAT6	0.39	0	_0.15	0.2	0.445042076	2.73E-27	0.439631175	3.29E-23
	STAT5A	0.3	2.5e_12	_0.12	0.33	0.164142675	0.000143792	0.212443513	4.19E-06
	CCR3	0.07	0.11	_0.47	3.6e_05	0.092586099	0.032590686	0.119407183	0.010287671
Th9	TGFBR2	0.53	0	_0.35	0.0023	0.570444531	2.47E-47	0.575117251	6.03E-42
	IRF4	_0.012	0.79	_0.38	0.0011	-0.010470555	0.809422873	0.021913167	0.638873321

SPI1 0.0025 0.95 _0.44 0.00013 -0.103505068 0.016863305 -0.084045274 0.071416452

Mast cells TPSB2 0.081 0.066 _0.33 0.0049 0.107057982 0.013400909 0.1127555190.015430264

TPSAB1 0.12 0.0048 _0.33 0.0043 0.124422641 0.004037173 0.134413256 0.003836873

CPA3 0.34 2e_15 _0.41 0.00039 0.392975107 0 0.395986574 9.30E-19

MS4A2 0.35 0 _0.38 0.001 0.338923363 8.58E-16 0.340790796 5.33E-14

HDC 0.091 0.038 _0.36 0.002 0.169135528 8.71E-05 0.209640171 5.64E-06

NK cells XCL1 _0.14 0.0011 _0.31 0.0088 -0.240556471 1.87E-08 -0.225994952 9.45E-07

CD7 _0.062 0.16 _0.55 7.1e_07 -0.318282293 6.94E-14 -0.311718395 7.59E-12

KIR3DL1 0.110.014 _0.34 0.0035 0.106478443 0.013914238 0.104623654 0.024676173

CD4+ T cell CD4 0.26 2.7e_09 _0.45 6.5e_05 0.130858879 0.002486878 0.147795618 0.001461689

M1 macrophage NOS2 0.18 5.4e_05 _0.089 0.46 0.196304681 0.082936303 0.277278678 0.017551262

IRF5 0.12 0.0057 0.15 0.21 -0.160881207 0.156390645 -0.054494779 0.647018617

PTGS2 0.0088 0.84 _0.045 0.71 0.218724896 0.052794104 0.292734469 0.011961646

M2 macrophage CD16 0.21 1.6e_06 _0.35 0.0023 -0.01285297 0.910363182 0.151078469 0.202000243

MRC1 0.47 0 _0.28 0.018 0.040895813 0.719935642 0.218551654 0.063225555

ARG1 _0.0075 0.86 _0.3 0.011 0.227324894 0.043929453 0.220353968 0.061025952

MS4A4A 0.28 1.3e_10 _0.39 0.00081 -0.030623174 0.788377656 0.164010095 0.165586435

Dendritic cell	THBD	0.19	7.8e_06	_0.28	0.016	0.075170399	0.509495854	0.202715519	0.085429587
	CD1C	0.21	9.7e_07	_0.42	0.00027	-0.019029794	0.867795285	0.132035817	0.26548217
	ITGAX	0.013	0.77	_0.41	0.00038	-0.085978578	0.450411827	0.073959586	0.534035972
Neutrophil	CEACAM8	0.13	0.0022	0.014	0.9	-0.272082377	0.015277745	-0.287772977	0.013558168
	ITGAM	0.11	0.0098	_0.37	0.0015	-0.014727361	0.897345583	0.188048475	0.111114209
	FUT4	0.43	0	_0.44	9.9e_05	0.301557936	0.007115432	0.268752656	0.021501253
TAM	CCL2	0.063	0.15	_0.22	0.062	0.098174294	0.38859654	0.257627092	0.027774019
	CD80	0.11	0.01	_0.24	0.046	-0.074643322	0.51325192	-0.012772522	0.914591214
	CCR5	0.15	0.00061	_0.41	0.00035	-0.091528724	0.421625151	0.029653805	0.803326755
	CD86	0.25	5.2e_09	_0.4	0.00042	-0.138485881	0.223058416	0.012406483	0.917030108
Tfh	BCL6	0.21	1.2e_06	0.043	0.72	0.20912853	0.064487334	0.189052195	0.109185416
	CXCR5	_0.49	3.7e_33	_0.43	0.00019	0.038538378	0.735964526	0.108766694	0.359674832
	ICOS	0.094	0.031	_0.44	9.1e_05	-0.126567513	0.26635204	0.009953508	0.933392726
Th17	STAT3	0.61	0	0.063	0.6	0.621908471	6.68E-10	0.654773924	3.30E-10
	IL-23R	0.057	0.2	_0.28	0.017	0.381230375	0.000527621	0.337595878	0.003490102
	IL-21R	0.1	0.021	_0.54	8.2e_07	0.179452223	0.113544949	0.241061335	0.039928831
	IL17A	_0.03	0.5	_0.18	0.14	0.111475595	0.32803641	0.177376183	

0.133283738

Th22	CCR10	_0.075	0.088	_0.056	0.64	0.231110029	0.040654651	0.249755384	0.033091089
	AHR	0.52	0	_0.24	0.046	0.252336904	0.025114732	0.321527308	0.005540912
Treg	FOXP3	_0.16	0.00027	_0.17	0.15	0.010223953	0.928660881	0.002828683	0.981051082
	CCR8	0.097	0.026	_0.36	0.0017	0.03871926	0.734770952	0.129246542	0.275800427
	IL2RA	0.086	0.049	_0.46	3.9e_05	0.214594688	0.057545206	0.405026432	0.000378794
T cell exhaustion	PD-1	_0.05	0.25	_0.43	0.00017	-0.238250635	0.034479822	-0.153703778	0.19418096
	CTLA4	_0.019	0.67	_0.29	0.013	-0.184589714	0.103409154	-0.077528709	0.514430231
	LAG3	_0.046	0.29	_0.061	0.61	-0.249683544	0.026726348	-0.226437159	0.054055715
	HAVCR2	0.25	7.6e_09	_0.46	4.7e_05	-0.003700097	0.9742068	0.171709314	0.14634144
Monocyte	CD14	0.022	0.71	_0.32	0.0066	-0.075632911	0.506888194	0.097260847	0.413016077
	FCGR3B	0.056	0.35	_0.23	0.049	0.106691138	0.34934975	0.235411807	0.044971953
	CSF1R	0.097	0.1	_0.34	0.0032	-0.141285297	0.213774155	0.024438251	0.837394559
